# Functional biomarkers that distinguish between tinnitus with and without hyperacusis

**DOI:** 10.1002/ctm2.378

**Published:** 2021-05-21

**Authors:** Benedikt Hofmeier, Jakob Wertz, Fatma Refat, Pauline Hinrichs, Jörg Saemisch, Wibke Singer, Lukas Rüttiger, Uwe Klose, Marlies Knipper, Stephan Wolpert

**Affiliations:** ^1^ Department of Otolaryngology Head and Neck Surgery Hearing Research Centre Tübingen University of Tübingen Tübingen Germany; ^2^ Department of Otolaryngology Audio‐Vestibular Medicine Unit Minia University Minya Egypt; ^3^ Department of Diagnostic and Interventional Neuroradiology University Hospital Tübingen Tübingen Germany; ^4^ Department of Otolaryngology Head and Neck Surgery Hearing Research Centre Tübingen Tübingen Germany

To the Editor:

We recently observed that tinnitus is associated with reduced auditory input that fails to increase neural gain due to diminished stimulus‐evoked responses.^1^, ^2^, ^3^ This was in contrast to views that suggested a homeostatic increase in neural gain to generate central hyper‐excitability leading to tinnitus.^4^ A curative therapy for tinnitus currently does not exist. Its progress is mostly impeded by the existing controversial views about the neural correlate of tinnitus that, depending on predictions, either would require the suppression or the enhancement of brain activity. We hypothesized that different neural correlates of tinnitus, whether with or without the co‐occurrence of hyperacusis, contributed to this dilemma. To test this hypothesis, we recruited 43 controls and 50 audiologically examined tinnitus patients with and without a co‐occurrence of hyperacusis (Tables S1 and S2) and performed brainstem audiometry (ABR) and functional imaging of brain activity (fMRI).

Among the group of 50 tinnitus patients, 20 could be identified with the co‐occurrence of hyperacusis (T+H group) from the HKI hyperacusis questionnaire (Figure 1A).^5^ The overall score of the Goebel and Hiller Score (G‐H‐S) tinnitus questionnaire^6^ was significantly higher for the T+H group than the tinnitus‐only patients (T group) (Figure 1B, *p *< .001***) for nearly all subscores (Figure S1, *p *< .002***). In the T+H group (Figure 1D) but not the T group (Figure 1C), auditory perceptional difficulty became worse for patients with self‐rated tinnitus loudness ≤15 dB HL (Figure 1E, Figure S2). The T and T+H group differences in annoyance and distress were not linked to differences in hearing sensitivity (Figure S3A–C, *p *> .5), since pure tone audiometry (PTA) thresholds (Supporting Material^1^) were not different between groups. In contrast, supra‐threshold ABR by brainstem‐evoked response audiometry (BERA)^1^, ^2^ revealed group differences: In T group, significantly reduced ABR wave V amplitude together with significantly prolonged interpeak latency (IPL) I–V (Figure 1F,H,J; Table S3) and reduced ABR wave V/I ratios (Figure 1I), were found. In contrast, T+H group showed a significantly higher ABR wave III and wave V amplitude at 75 dB compared to controls (Figure 1G,H; Table S3) with no difference in ABR wave V/I ratio (Figure 1I). Questioning if these group differences were reflected in BOLD fMRI responses, stimulus‐induced BOLD fMRI signals were recorded from anatomically predefined ROIs in ascending auditory regions (Table S4) in response to binaurally exposed (i) rock music, (ii) LF‐chirp, (iii) HF‐chirp, and (iv) BB‐chirp stimuli (Figure 2).

**FIGURE 1:**
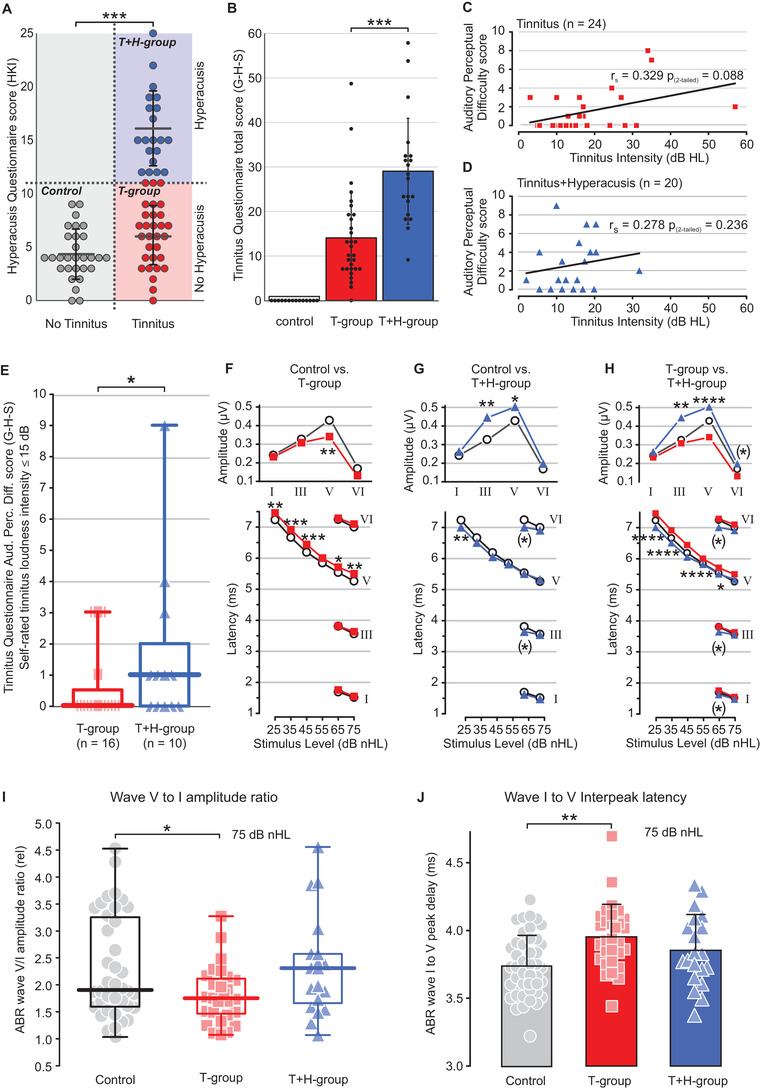
Hyperacusis Questionnaire total score (HKI) and Tinnitus Questionnaire total score (G‐H‐S). The figures represent mean ± SD between control (*n *= 29, gray), T group (*n *= 30, red), and T+H group (*n *= 20, blue) for (A) HKI score, and (B) Tinnitus Questionnaire total score (G‐H‐S) (see Supporting Material Methods). Mann–Whitney *U*‐test was used to calculate the group differences. Two‐tailed Spearman correlation of tinnitus loudness with the Tinnitus Questionnaire subscore “auditory perceptual difficulties” for individual participants: (C) T group (red), and (D) T+H group (blue). The box plot (E) shows median, range (whiskers), and quartiles (box) of the Tinnitus Questionnaire Auditory Perceptual Difficulty (Aud. Perc. Diff. score) for patients with self‐rated tinnitus loudness intensity ≤15 dB HL. In the T+H group (D) but not the T group (C), the auditory perceptional difficulty correlated quite well with the self‐rated tinnitus intensity (for the correlations of all subscores see Figure S2), due to an auditory perceptional difficulty that in the T+H group became particularly annoying for self‐rated tinnitus when loudness was ≤15 dB HL (E). Mann–Whitney *U*‐test was used to calculate the group differences between T group (*n *= 16, red) and T+H group (*n *= 10, blue). (F) ABR wave amplitude changes and latency shifts. Averaged ABR wave I, III, V, and VI amplitudes at 75 dB nHL (upper panels), and latency as function of stimulus level (lower panels). Lines connect data points of the same experimental group. Repeated measures two‐way ANOVA was used to test for group differences within single ABR waves and Holm–Sidak's multiple comparison test for pairwise differences between control (*n *= 43, gray) and T group (*n *= 30, red), (G) between control and T+H group (*n *= 20, blue), and (H) between T group and T+H group. For wave I, III, and VI, distinct responses were limited to 65 and 75 dB nHL stimuli. The box plots (I) show median, quartiles, and range of ABR wave ratio V/I at 75 dB nHL for control (*n *= 43, gray), T group (*n *= 30, red), and T+H group (*n *= 20, blue). Interpeak latency (IPL) between wave I and wave V peak is shown in the bar graphs (J) as mean ± SD. Details of statistical results are given in Table S3. The overall differences that emerged from the Tinnitus Questionnaire (G‐H‐S) for the T and the T+H groups were not related to gender (Table S3), left‐ or right‐handedness of the participants, tinnitus laterality, tinnitus intensity, tinnitus frequency, nor with the age of the participants (Table S1). The reduced ABR wave V responses in the T group were reflected in reduced ABR wave V/I ratio (Figure 1I), confirming assumptions of reduced central neural gain with elevated response variability in tinnitus.^9^ *p < .05, **p < .01, ***p < .001, ****p < .0001 from Holm–Sidak's multiple comparison test; (*) statistical test close to statistical significance (*p* < .066 in Holm–Sidak's multiple comparison test). ABR, auditory brainstem response; Aud. Perc. Diff., auditory perceptual difficulties; dB, decibel; G‐H‐S, Goebel and Hiller Score; HL, hearing level; nHL, normalized hearing level; RM, repeated measures two‐way ANOVA; SD, standard deviation

**FIGURE 2:**
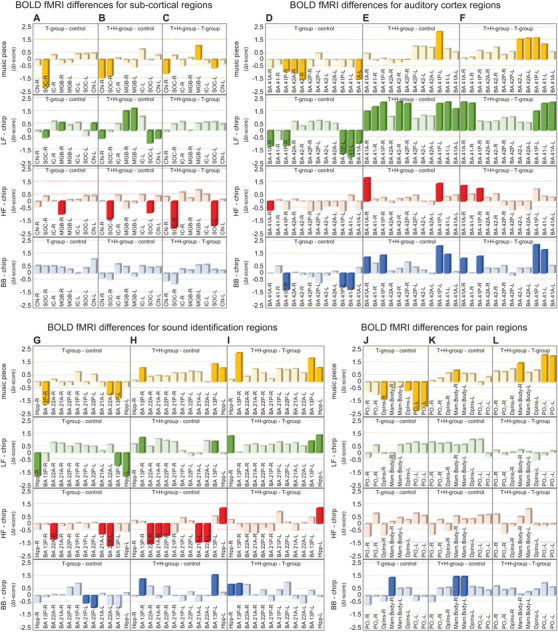
Task evoked fMRI group differences for subcortical regions (A–C), auditory cortex regions (D‐F), sound identification‐associated regions (G–I), and pain‐associated regions (J–L). Differences for significant (two‐sample *t*‐test, *p *< .05, false discovery rate corrected) task evoked BOLD activity (reduced or enhanced as Δ*t* score compared to the respective group notified within each panel) for the predefined brain areas (Supporting Material, Methods and Table S4; predefined ROIs for taskevoked and resting‐state fMRI). Control (*n *= 43), T group (*n *= 30), and T+H group (*n *= 20). First row: music piece; second row: low‐frequency (LF) chirp; third row: high‐frequency (HF) chirp; last row: broadband (BB) chirp. A, anterior; BA, Brodmann area; CN, cochlear nucleus; DpIns, dorsal posterior insula; FDR, false discovery rate; Hipp, hippocampus; IC, inferior colliculus; L, left; MGB, medial geniculate body; P, posterior; PO, parietal operculum; R, right; ROI, region of interest; SOC, superior olivary complex

A significant reduction in BOLD fMRI signals in lower auditory brainstem regions (SOC, partly CN) (Figure 2A–C; music, LF stimuli) revealed as a characteristic feature of tinnitus in both groups.

From the MGB upwards, BOLD fMRI signals between groups differed, remaining reduced in the T group in the MGB (Figure 2A,C; music, HF), primary auditory cortex (AC‐I) (Figure 2D,F; all stimuli), and regions of sound identification (Figure 2G,I; all stimuli), but become elevated in the T+H group in the MGB (Figure 2B,C; music, LF), AC‐I (Figure 2E,F; all stimuli), or regions of sound identification in the T+H group (Figure 2G,J; music, LF, BB). Regions active immediately following painful stimulation,^7^ such as the mammillary body (Mam. Body), the dorsal posterior insula (DpIns), and the postcentral gyrus regions with the parietal operculum (PO_1_, PO_2_) responded with reduced BOLD fMRI signals to music stimuli in the T group, but not in the T+H group (Figure 2J,L; music), suggesting more response activity in pain regions to sound in the T+H group. Interested if evoked BOLD fMRI responses were related to BOLD signals at rest, as hypothesized,^8^ we strikingly observed that the number of correlations of BOLD signals at rest (r‐fcMRI) (Figure 3C–L), but not the correlation strength (Figure 3B), when depicted as positive or negative correlations (Figure 3A, Figure 3D‐L, lower panel) were related to altered evoked BOLD signals between groups. The number of connectivities between the MGB and subcortical auditory regions such as the CN, SOC, IC (Figure 3D), between MGB and the anterior AC‐I regions BA41 and BA42 (Figure 3E), between the AC‐I and regions controlling emotional distress, particularly the amygdala (Figure 3G), and between the AC‐I and attention‐controlling regions such as BA45 and BA46 (Figure 3H) was significantly lower in the T group compared to the T+H group. Few regions with negative correlations that were lower in the T group and T+H group may need further future specification.

**FIGURE 3:**
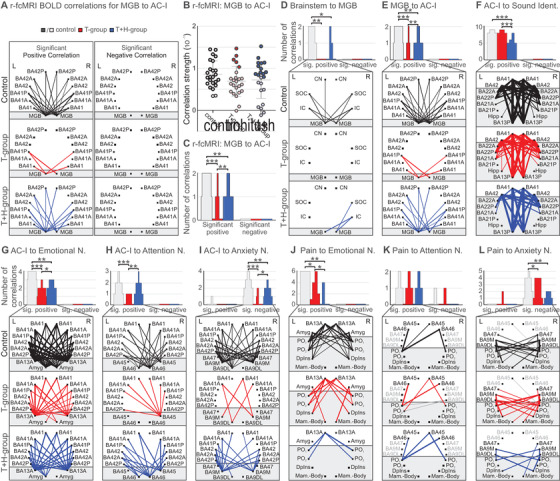
3Illustration of methods for evaluating qualitative and quantitative group differences in r‐fcMRI correlations of the ascending auditory pathway, auditory cortex networks, and pain networks. The patterns in (A) show the amount of significant nonzero (one‐sample *t*‐test *p *< .05, FDR corrected) r‐fcMRI BOLD correlations between predefined ROI groups of the scatterplot (B), divided into positive and negative correlations for the different groups. In the scatterplot (B), each data point represents the group mean of the correlation strength between two coordinates of the considered predefined ROI groups (in this example MGB and the AC‐I [BA41; BA41A; BA41P; BA42; BA42A; BA42P]). Mean ± SD shown for control (*n *= 43, gray), T group (*n *= 30, red), and T+H group (*n *= 20, blue). Significantly nonzero values are highlighted. The bar chart (C) shows the amount of significant nonzero r‐fcMRI BOLD correlations of figure (A). The thickness of lines shown in the lower column corresponds to the correlation strength. For the group comparisons, the data are Aligned and Rank Transformed (ARTool) and the variance is determined with repeated measures ANOVA. The graphs (D–F) show the amount of significant nonzero (one‐sample *t*‐test *p *< .05, FDR corrected) r‐fcMRI BOLD correlations between predefined ROI groups, divided into positive correlations and negative correlations, for the different groups. Control (*n *= 43, gray/black), T group (*n *= 30, red), and T+H group (*n *= 20, blue). For group comparisons, the data are Aligned and Rank Transformed (ARTool) and the variance determined with a repeated measures ANOVA (see Supporting Material Methods). Connectivities between (D) lower brainstem (CN, SOC, IC) and the MGB, (E) MGB and the AC‐I (BA41; BA41A; BA41P; BA42; BA42A; BA42P), (F) AC‐I (for better clarity without A/P) and the Sound Identification Network (BA21A; BA21P; BA22A; BA22P; Hipp; BA13P), (G) AC‐I (BA41; BA41A; BA41P; BA42; BA42A; BA42P) and the Emotional Distress Network (BA13A; Amyg), (H) AC‐I and the Attention Network (BA45; BA46), (I) AC‐I and the Anxiety Network (BA47; BA9M; BA9DL), (J) pain‐associated ROIs (PO_1_; PO_2_; DpIns; Mam. Body) and the Emotional Distress Network, (K) Pain Network and the Attention Network, and (L) Pain Network and Anxiety Network. As a most characteristic sign, the number of correlations (C–L), but not the correlation strength (B) of the interregional connections, was analyzed. AC‐I, auditory cortex; Amyg, amygdala; BA, Brodmann area; BOLD, blood oxygenation level depended; CN, cochlear nucleus; DL, dorsolateral; DpIns, dorsal posterior insula; FDR, false discovery rate; Hipp, hippocampus; IC, inferior colliculus; M, medial; Mam, mammillary; MGB, medial geniculate body; PO, parietal operculum; ROI, region of interest; SOC, superior olivary complex

To summarize, as a most characteristic functional biomarker of the T group, the present study identified (i) delayed and reduced ABR wave V; (ii) reduced evoked BOLD fMRI responses in the MGB, AC‐I, and regions of sound identification as the BA13P and hippocampus, particularly specific in response to HF‐chirp stimuli; and (iii) reduced number of positive connectivities between subcortical and cortical auditory regions (Figure 4, red). As a characteristic functional biomarker for tinnitus with a co‐occurrence of hyperacusis (i) enhanced ABR wave III and ABR wave V for high sound intensities; (ii) elevated evoked BOLD fMRI responses in the MGB, AC‐I, BA13P, and hippocampus particularly for LF‐chirp stimuli; and (iii) greater number of positive connectivities between subcortical and cortical auditory regions compared to the T group (Figure 4, blue) were found. Group differences were independent of G‐H‐S group differences (Supporting Material). We conclude that the overall reduced and delayed auditory‐specific responsiveness in the T group is best corroborated by previous assumption of a loss of fast (high‐SR) auditory fiber processing in tinnitus frequency channels leading to re‐emergence of hyperexcitability through loss of tonic parvalbumin interneuron in deprived regions.^9^ This would lead to diminution of memory‐linked contrast amplification and elevated noise, and as a result would promote further alertness and attention to the phantom noise, as reviewed.^9^ With the co‐occurrence of hyperacusis, a more widespread signal amplification process appears to proceed through overactive thalamo‐cortical activity that may trigger an excitation spread to limbic and pain regions, and results in overattention to increased loudness at all sound frequencies, as was also previously hypothesized.^10^ The findings may eventually lead to new differential clinical diagnosis of tinnitus, a prerequisite for achieving a successful, personalized curative therapy for tinnitus with and without hyperacusis, when regarding suggestions for altered strategies to find treatment predictors.^9^

**FIGURE 4:**
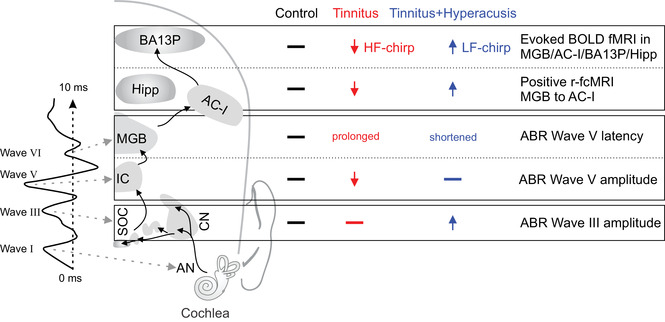
4Overview for the characteristic functional biomarkers discriminating the T and T+H groups (r‐fcMRI, evoked fMRI, ABR wave amplitude and latency). Wave I, III, V, VI distinct ABR wave components. Horizontal bar: unchanged; down arrow: smaller; Up arrow: larger/more. ABR, auditory brain response; AC‐I, auditory cortex; AN, auditory nerve; BA, Brodmann area; BOLD, blood oxygenation level‐dependent; CN, cochlear nucleus; Hipp, hippocampus; IC, inferior colliculus; LF, low frequency; MGB, medial geniculate body; SOC, superior olivary complex

## CONFLICT OF INTEREST

The authors declare that there is no conflict of interest that could be perceived as prejudicing the impartiality of the research reported.

## ETHICS APPROVAL AND CONSENT TO PARTICIPATE

The ethics committee of Tübingen University (Faculty of Medicine) and University Hospital Tübingen (ethical approval number 264‐2016BO1) approved this study. Trial registration: German Clinical Trials Register DRKS0006332. Written informed consent was obtained from all participants at their first visit.

## AUTHOR CONTRIBUTIONS

Designed research: Marlies Knipper, Lukas Rüttiger, Uwe Klose, and Stephan Wolpert. Conducting experiments: Benedikt Hofmeier, Jakob Wertz, Fatma Refat, and Pauline Hinrichs. Analyzed data: Benedikt Hofmeier, Jakob Wertz, Fatma Refat, Pauline Hinrichs, Jörg Saemisch, Wibke Singer, and Lukas Rüttiger. Wrote the first draft of the paper: Marlies Knipper and Uwe Klose. Edited the paper: Marlies Knipper, Lukas Rüttiger, Uwe Klose, and Stephan Wolpert. Wrote the paper: Benedikt Hofmeier, Jakob Wertz, Lukas Rüttiger, Uwe Klose, Stephan Wolpert, and Marlies Knipper.

## CONSENT FOR PUBLICATION

All the authors have read the manuscript and approved its submission to Clinical and Translational Medicine.

## DATA AVAILABILITY STATEMENT

The data that support the findings of this study are available from Marlies Knipper upon reasonable request.

## Supporting information

Supporting Information
