# Supplementary material for: Functional biomarkers that distinguish between tinnitus with and without hyperacusis
Source: Clin Transl Med. 2021 May 21;11(5):e378. doi: 10.1002/ctm2.378 (PMC8140185; doi:10.1002/ctm2.378)
Supplement: Supplementary file 1 — Supporting Information [file CTM2-11-e378-s001.pdf]

MATERIALS AND METHODS

Participants

The study was approved by the ethic committee of Tübingen (Trial Registration: 391/2018BO2). A total of 93 participants were included in the present trial. Hearing thresholds did not exceed 20 dB at each frequency from 0.125 kHz to 3 kHz and not 40 dB at each frequency from 4 kHz to 10 kHz in the pure tone audiometry (PTA). Handedness, Age and Gender were regarded (Supplementary Table 1, 2) to compare homogeneity of the groups. 50 tinnitus patients were recruited for sub-classification as either T-group (mean age 29.73 ± 7.86 years, age range 20-50 years) or T+H-group (mean age 26.95 ± 6.94 years, age range 18-57 years) based on the Hyperacusis inventory (HKI) score, and 43 subjects (mean age 26.51 ± 5.83 years, age range 18-45 years) without tinnitus and hyperacusis could be included in the control group.

Age, gender and handedness relation to results

Chi-square tests of independence showed that there were no significant group differences regarding handedness ($\chi 2$ (6, n = 93) = 8.7207, p = 0.19) and age (χ 2 (3, n = 93) = 3.6154, p = 0.46); divided in to three groups: ≤ 23, ≥ 28 and in between). Group differences were significant for gender ($\chi 2$ (2, n = 93) = 8.1995, p = 0.017). Since ABR amplitudes are higher in women than in men[1] we inspected wave V amplitude and latency for women and men separately. No significant differences in wave V amplitude and latency (75 dB nHL) were observed (see Supplementary Table 3 ABR Wave V gender comparison).

Hyperacusis questionnaire

In order to assess the presence of hyperacusis and to differentiate it from phonophobia/recruitment a Hyperacusis Questionnaire (HKI)[2] was administered to all participants in all groups. The HKI was developed based on the “Hyperacusis Questionnaire” (HQ)[3], and of the GFÜ[4] and consists of 9 statements which are evaluated with a score from 0 to 2. When subjects exceeded a score > 11, they were considered as hyperacusis patients.[2]

Tinnitus questionnaire

The Goebel-Hiller-Score (G-H-S) tinnitus questionnaire with several questionnaire sub-scores in addition to the total tinnitus score was used to assess different aspects concerning tinnitus severity, laterality, emotional distress, cognitive distress, self-experienced intrusiveness, and auditory perceptual difficulty scores.[5-7] All subjects with tinnitus were asked to answer 52 statements in the questionnaire for different conditions that have impact on their lives, and they

were asked to answer if this statement is true, partially true or not true. The different questionnaire scores were collected and every sub-score calculated according to the answers of the probands with tinnitus. The analyzed scores were then correlated with tinnitus loudness levels of all tinnitus participants to find out which aspects are related to tinnitus intensity measured during audiological evaluation.

G-H-S score relation to ABR and BOLD fMRI

As suggested by the reviewer, we defined subgroups (n = 8) from the T- and TH-group with a similar tinnitus severity (G-H-S total score in the range of 15 to 25). These subgroups were reanalyzed for suprathreshold/ latency ABR wave differences and for task evoked fMRI. For the reviewer's information we here provide selected results for these subgroups, that are now newly provided as information to the Supplementary Material (It is written on page 2: G-H-S score relation to ABR and BOLD fMRI) and following changes in the Letter:

Repeated measures two-way ANOVA was used to test for group differences within ABR wave amplitudes ($F(1, 14) = 8.75$, $p = 0.0104$) and latencies ($F(1, 14) = 9.036$, $p = 0.0094$). Holm-Sidak's multiple comparison test showed significant group differences ($p = 0.0067$) between control (mean = 0.42 ± 0.17, n = 43), T-group (mean = 0.35 ± 0.11, n = 8) and T+H-group (mean = 0.54 ± 0.19, n = 8) for amplitude V (75 dB). The statistical outcome therefore mirrors the group differences in Figure 1 F-H (although with less statistical power due to smaller sample size).

For the verification of the fMRI task evoked analysis, the BOLD activity of the primary auditory cortex regions (BA41) was calculated for LF- and HF-chirp and compared between T- and TH-group of similar G-H-S scores (two-sample t-test, false discovery rate corrected). For the HF-chirp, a significant increase was found in the right posterior region BA41P in the TH-group compared to the T-group ($\Delta t = 1.15$, $p = 0.04$). For the BB-chirp, a significant increase was found in the right anterior region BA41A in the TH-group compared to the T-group ($\Delta t = 1.89$, $p = 0.03$).

In conclusion: The comparison of group differences between a smaller group size of participants with T and T+H but similar G-H-S scores, confirmed the findings of larger group sizes with different G-H-S scores, as here demonstrated for amplitude and latency of ABR wave V and evoked BOLD responses in selected regions. This suggests that tinnitus severity itself is not the decisive factor for differences in brain activity and stimulus responses.

Audiological evaluation

Ear examination, pure tone audiometry, speech audiometry, tympanometry and, acoustic reflex measurements were determined as described in.[8]

PTA thresholds were measured from 0.125 to 10 kHz (0.125; 0.25; 0.5; 1; 1.5; 2; 3; 4; 6; 8 and 10 kHz), for speech audiometry the German monosyllabic "Freiburger Test" was performed (AT 900 Audiometer, Auritec, Medizindiagnostische Systeme GmbH, Hamburg, Germany).

Acoustic reflex measurements were performed at four frequencies (0.5, 1, 2, and 4 kHz) from 80 to 100 dB sound pressure level in 5 dB steps using the same device as for tympanometry (Madsen-Zodiac 901, GN Otometrics, Münster, Germany).

Auditory brainstem potentials

ABR was tested with a GSI Audera device (Grason-Stadler, Eden Prairie, USA) using Telephonics TDH 39p headphones (Telephonics, Farmingdale, USA) and Neuroline 720 skin electrodes (Ambu, Bad Nauheim, Germany). Details are described in Hofmeier et al. (2018). In short, the pre-amplified and band-pass filtered (150-3000 Hz) brainstem potentials evoked by broadband acoustic clicks (0.1 ms duration) were recorded for 10 ms post-stimulus time and 2000 repetitions at stimulus levels between 25 and 75 dB normalized hearing level in 10 dB steps.

Calculation of supra-threshold ABR wave fine structure

From the ABRs averaged for 2000 repetitions separately for each stimulus level and ear the single wave components at defined latencies were analyzed for amplitudes of deflections (peaks) and peak times where consecutive positive and negative peaks result in a potential-wave (wave I, III, V, and VI for time intervals at 1-2 ms, 3-4 ms, 5-6 ms, and 6-7 ms, respectively). In addition, the inter-wave latencies I-III, III-V and I-V were analyzed. The data for all the supra-threshold amplitudes, wave latencies and inter-wave latencies were analyzed for individual ears and the averaged for experimental groups for presentation and statistical analysis.

Functional magnetic resonance imaging (fMRI)

fMRI image acquisition was performed on a 3-Tesla scanner (Prisma Fit, Siemens, Germany). For the acoustic stimulation, we used special MRI-suitable over-ear headphones (CONFON HP-SC 03, MR Confon GmbH, Magdeburg, Germany). During scanning, four different auditory stimuli were generated using a stimulus presentation software (Neurobehavioral

Systems software, Neurobs, Berkeley, USA; Panasonic-SC-PMX5 Amplifier, Panasonic Marketing Europe GmbH, Hamburg, Germany).

The measurement experimental design of the task fMRI

The task-evoked functional images were obtained with a T2* weighted echo-planar sequence. 155 volume data sets were acquired (TE 35 ms, matrix 64*64 slice thickness 3 mm, slice gap 0.75 mm 30 slices). The repetition time (TR) between acquisitions was approximately 2 s and defined by a trigger pulse from a finger plethysmograph. This cardiac gating was used to avoid signal distortion due to brain pulsation induced by the heart function especially near the brainstem. Here, we used a specific TR-correction procedure, aiming to eliminate the signal variation due to variable TRs that would obscure the BOLD signal.[9] The TR correction was based on a T1 estimation in each pixel evaluating signals from all 155 measurements which were obtained with variable TR values. A hypothetic signal intensity for a fixed TR of 2 s was calculated. BOLD fMRI activity was calculated in regions that were defined prior to the fMRI using anatomical datasets (Supplementary Table 4). Aiming at measuring the BOLD signal in response to different acoustic stimuli, the presenter software version 16.1 (Neurobehavioral Systems software) was used to present four different acoustic stimuli with a specially written protocol. Stimuli were applied in 7 blocks with a length of about 20 s each. Each block was applied for 10 measurements, the exact length of each block depended on the cardiac pulse interval length of the examined subject. After each stimulus block, 10 measurements were acquired without any acoustical input.

The stimuli used are (1) high-frequency chirp (HF-chirp), (2) low-frequency chirp (LF-chirp), (3) broadband chirp (BB-chirp) and (4) a rock music piece. The chirps have a maximum power in the frequency range of HF-chirp (12 kHz to 20 kHz), LF-chirp (0.25 kHz to 3 kHz) and BB-chirp (0.3 kHz to 25 kHz). Task (evoked) fMRI analysis was performed as described in detail in.[8]

The measurement experimental design of the resting-state fMRI (r-fcMRI)

The resting-state functional images for the whole brain were acquired over a 10 minutes acquisition time period of awake rest. Measurement parameters beside TR (2 s) and number of slices (30) were identical to task evoked. To get particular information about the resting-state differences in lower brainstem (cochlear nucleus, superior olivary complex, inferior colliculus), the field of view block (normally aligned to anterior commissure – posterior commissure line) of the excited slices were moved toward lower brain regions. This technical adaptation is new in the investigation of the auditory system using r-fcMRI studies and was

first described in.[8] The participants were instructed to remain alert with their eyes closed, with no task to perform. Earplugs were used for all participants during the scan to reduce noise generated by the scanner. 300 volumes were acquired. Exact measurement parameters as well as r-fcMRI analysis are described in detail in.[8]

Statistics

For the statistical tests MATLAB programming system (version R2020a, MathWorks Inc., Natick, MA, USA), PRISM 8 (Graphpad) and R (open-source programming language) were used for evaluation. fMRI results are corrected for False Discovery Rate (FDR). Unless otherwise noted, statistical significance was tested at the level of $\alpha = 5$ %. Level of significance is illustrated in the figures with symbols or shaded areas (not significant (n.s.); $p > 0.05$; * $p \leq 0.05$; ** $p < 0.01$; *** $p < 0.001$).

For the hyperacusis questionnaire data are presented as mean and standard deviation (**SD**) for, T-group ($n = 30$), T+H-group ($n = 20$) and control ($n = 29$) (for age, sex and handedness see Supplementary Table 1). Shapiro-Wilk test was used to test scores of each group for normality of distribution; both groups were not normal distributed. Levene's Test confirmed equal distribution for both groups. Therefore, Mann-Whitney U test was used to test for differences in questionnaire total score between the two groups.

For the tinnitus questionnaire data are presented as mean and SD for, T-group ($n = 30$), T+H-group ($n = 20$) and control ($n = 43$). Scores were not normal but equally distributed. Mann-Whitney U test was used to test for differences in the questionnaire total score and sub-scores between the two groups (Supplementary Figure 1).

For each participant the Pearson correlation between the tinnitus score and loudness of tinnitus percept (dB hearing level) was calculated (Supplementary Figure 2). T-group, for the right and left ear ($n = 24$). T+H-group, for the right ($n = 17$) and left ($n = 19$) ear (for age, sex and handiness see Supplementary Table 1). Additionally, Mann-Whitney-U test was used to test for group differences in the (G-H-S) auditory perceptual difficulty score for patients with tinnitus intensity ≤ 15 dB hearing level in the T-group ($n = 16$) and T+H-group ($n = 10$).

For the pure tone audiometry (Supplementary Figure 3) data are presented as mean with SD for T-group ($n = 30$), T+H-group ($n = 20$) and control ($n = 43$) (for age, sex and handiness see Supplementary Table 1). The Shapiro-Wilk test was used to test each group for normal distribution and the Levene test for equal distribution, while the Mann-Whitney U test was

used to test for differences in hearing threshold between the two groups at frequencies ranging from (0.125 kHz to 10 kHz).

ABR wave amplitudes, latencies, and inter peak-latencies were determined for individual ears, and then averaged for left and right ear for each single participant. Wave V to I amplitude ratios smaller than 1 and larger than 5 were excluded from the analysis since these outliers misaligned the midpoints towards non-represented data ranges. Values from individual participants of each experimental group were inspected for normality of distribution by the Shapiro-Wilk test, and compared for significance of differences between group means by repeated measurement (RM) 2-way ANOVA.

For ROIs with reduced or enhanced evoked BOLD fMRI signal (Supplementary Table 4) data are presented as significant ($p < 0.05$, FDR corrected) difference in defined brain region activity in group comparison, for T-group ($n = 30$) and T+H-group ($n = 20$) and control (n = 43) (for age, sex and handiness see Supplementary Table 1). The second level specification independent two-sample *t*-test of the Statistical Parametric Mapping toolbox was used to perform the group analysis. Three different contrasts were calculated in the analysis. The first contrast is (tinnitus - control), in this case brain regions showing increased evoked response in the tinnitus group in comparison to the control group are represented. Accordingly, the second and third contrast are (tinnitus+hyperacusis - control) and (tinnitus+hyperacusis - tinnitus).

For connectivities between defined ROIs in r-fcMRI (Supplementary Table 4) data are presented as significant ($p < 0.05$, FDR corrected) positive or negative correlation coefficients, for T-group ($n = 29$), T+H-group ($n = 17$) and control ($n = 42$) (for age, sex and handiness see Supplementary Table 1). Data preprocessing steps were performed as described in [8].

After calculating the correlation values the correlations between ROIs of the different predefined areas (brainstem regions, primary auditory cortex, auditory identification network, emotional distress network, temporo-frontal attention network and the anxiety network) are extracted and analyzed.

In order to compare the number of connectivity's between areas of interest among the groups, the data are simplified. The correlation values between the ROIs are divided into three subgroups by a one-sample *t*-test (not significantly different from zero, significantly positive and significantly negative). To compare the connectivity between two areas in an analysis of variance, the number of significant correlations from each ROI of the first area to all ROIs of the second area is counted.

Due to the non-parametric and repeated measures data an align-and-rank transformation is performed with the ARTool[10] before the variances of the groups are analyzed. Afterwards a repeated measures analysis of variance of the aligned rank transformed data is performed in R using the ARTool library "*m <- art(sig_Corr ~ Group + Error(RoI), data = data)*". If the ANOVA shows a significant effect, a post hoc comparison is performed. The contrasts in ART within a single factor (Group – levels: control, T and T+H) may be computed with estimated marginal means (EMMs)/ least-squares means in R using the emmeans library „*emmeans(artlm(m, "Group"), pairwise ~ Group)*" (Confidence level used: 0.95; *p* value adjustment: tukey method for comparing a family of 3 estimates).

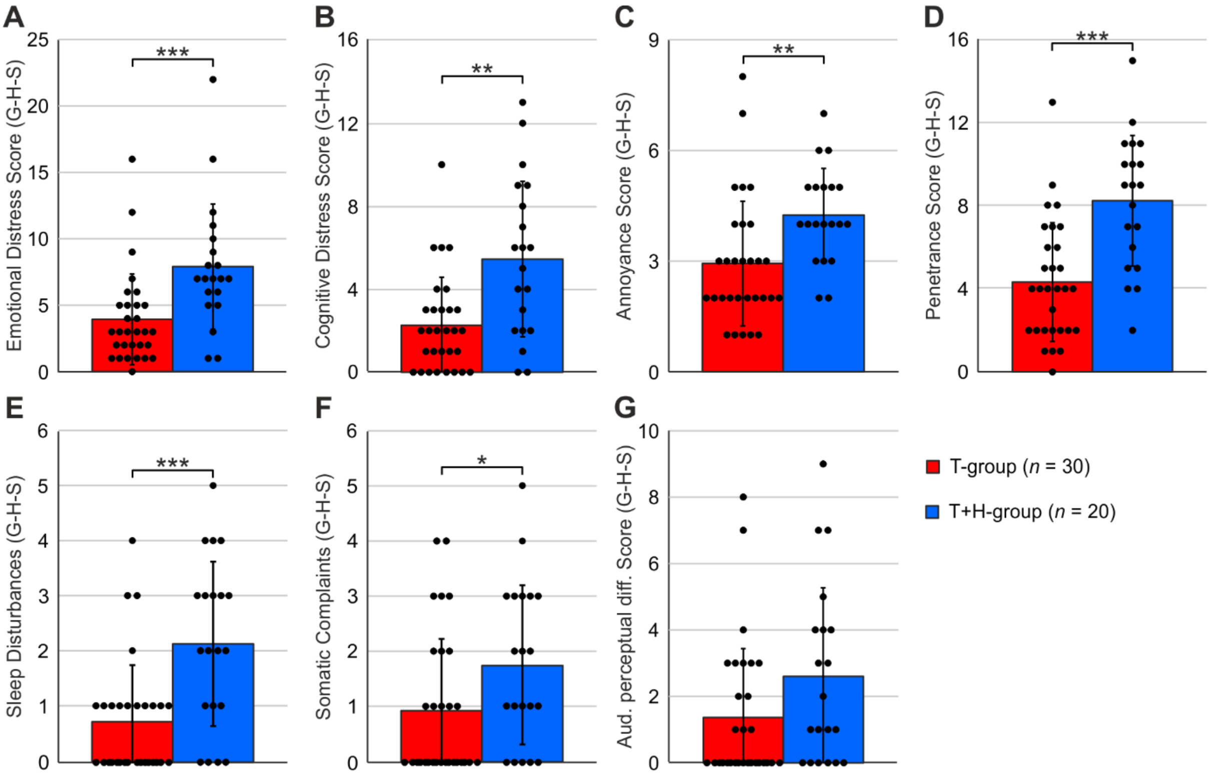

Supplementary Figure 1

Tinnitus questionnaire sub-scores. The bar charts illustrate the comparison of Tinnitus Questionnaire sub-scores mean ± SD between T-group (*n* = 30, red) and T+H-group (*n* = 20, blue). Mann-Whitney-U test was used to calculate the group differences. (G-H-S); Goebel and Hiller Score, SD; Standard Deviation.

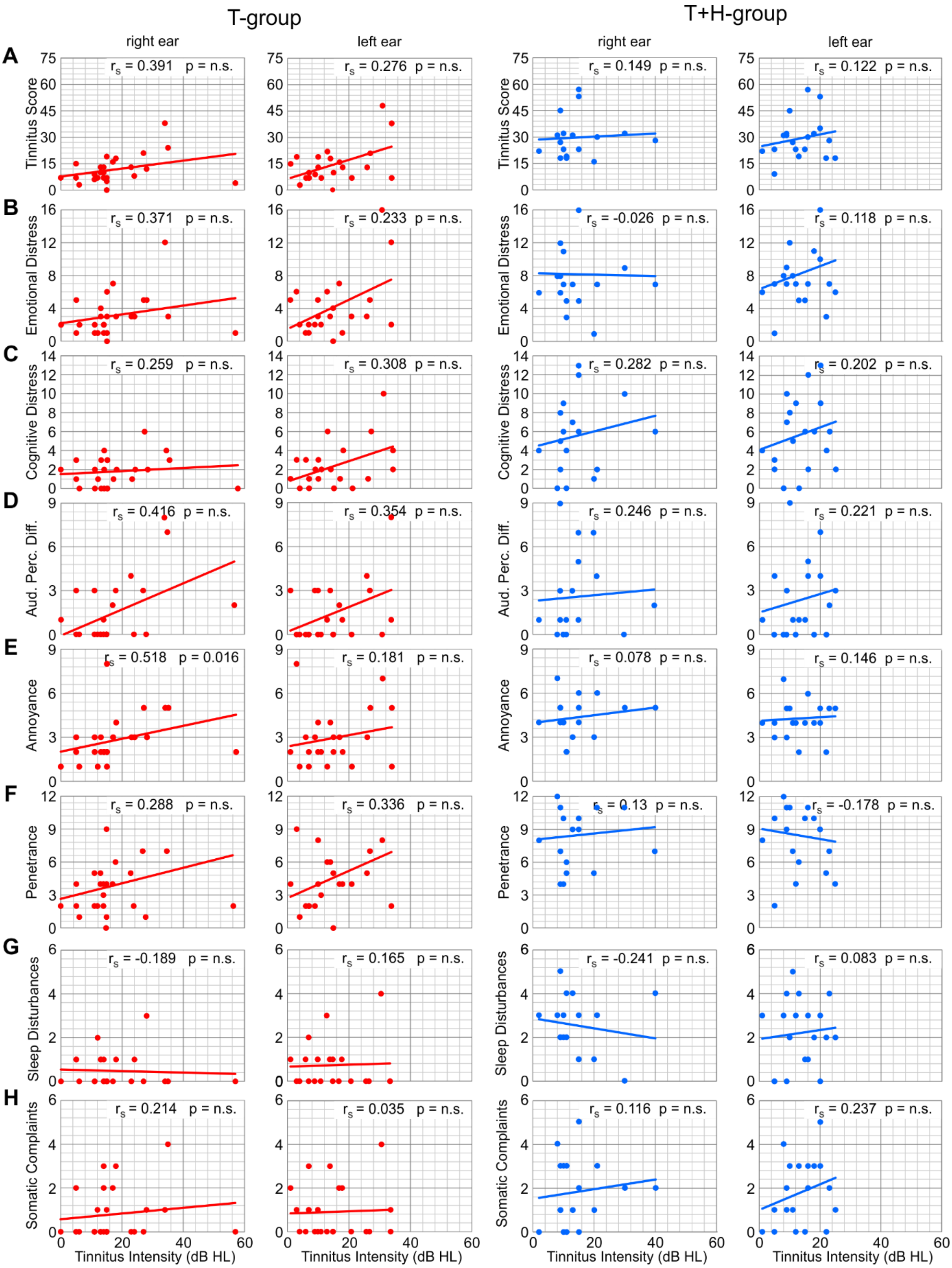

Supplementary Figure 2

The correlation between the sub-scores of the (G-H-S) and Tinnitus Loudness Intensity. Two-tailed Spearman correlation of tinnitus loudness (*n* depends on the perception of tinnitus in the respective ear) with Tinnitus-Questionnaire sub-scores (**A**-**H**) in T-group (*n* = 24 for the right and left ear, red) and T+H-group (*n* = 17 for the right ear, *n* = 19 for the left ear, blue). Adjusted p values by FDR correction for multiple testing. (**A**) Total Tinnitus score, (**B**) emotional distress, (**C**) cognitive distress, (**D**) auditory perceptual difficulties, (**E**) annoyance, (**F**) penetrance, (**G**) sleep disturbances and (**H**) somatic complaints.

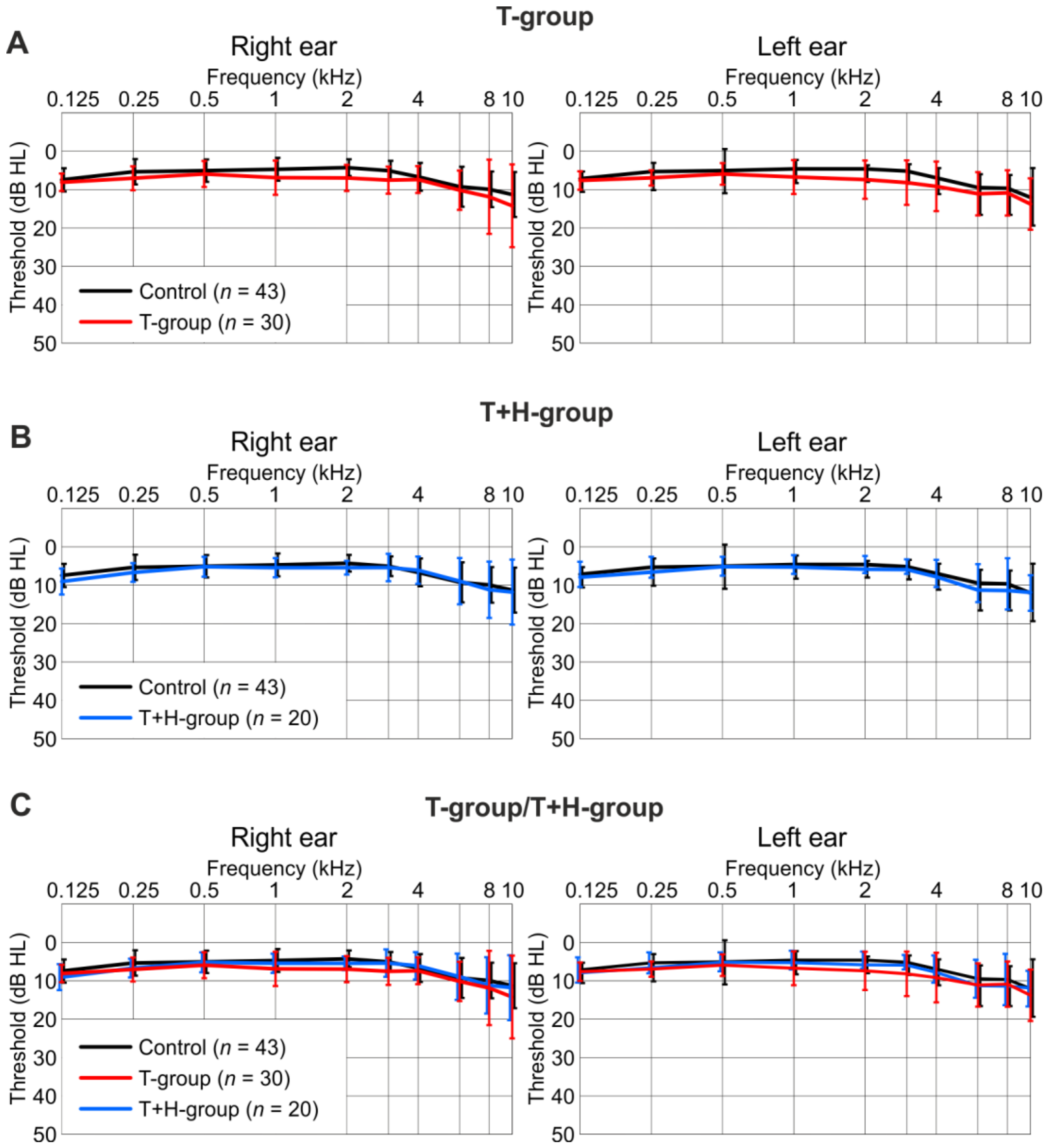

Supplementary Figure 3

Averaged pure tone audiometry (mean ± SD) for the control (*n* = 43, black), T-group (*n* = 30, red) and T+H-group (*n* = 20, blue), separated for the right and left ear. Mann-Whitney-U test was used to check for group differences. (**A**) T-group compared to control (**B**) T+H-group compared to control (**C**) T-group compared to T+H-group and control.

dB, decibel; HL, hearing level; kHz, kilohertz; SD, Standard Deviation.

Supplementary Table 1 (Study participants demographic data)

Groups											
Control				Tinnitus				Tinnitus+Hyperacusis			
Control	Age	Sex	Handedness	Tinnitus	Age	Sex	Handedness	Tinnitus+ Hyperacusis	Age	Sex	Handedness
K002	27	female	right	T001	36	male	left	TN12	24	male	right
K006	39	male	right	T002	21	male	right	TN13	21	female	left
KN01	21	female	right	T006	45	female	right	TN17	26	female	right
KN02	26	female	right	T009	34	male	right	TN19	34	female	right
KN03	18	male	right	TN01	26	male	right	TN20	28	female	right
KN04	32	female	right	TN03	34	male	left	TN21	24	female	right
KN05	41	female	left	TN04	23	male	left	TN22	21	female	right
KN06	21	female	right	TN05	33	male	right	TN23	24	female	right
KN07	23	female	right	TN08	27	male	right	TN25	22	female	right
KN08	18	female	right	TN10	25	female	right	TN28	30	female	right
KN09	19	male	right	TN11	25	female	right	TS020	24	female	right
KN10	20	female	right	TN16	26	male	left	TS033	49	male	left
KN11	24	female	right	TS004	44	male	right	TS037	23	female	right
KN14	20	female	right	TS005	29	male	right	TS040	23	female	right
KN16	27	male	left	TS008	20	female	right	TS044	20	male	left
KN17	24	male	left	TS010	26	male	right	TS048	27	male	right
KN18	26	male	right	TS017	29	male	right	TS050	21	female	right
KN20	27	male	right	TS019	25	male	right	TS057	33	male	right
KN21	28	male	right	TS021	27	male	right	TS061	29	male	right
KN22	26	male	right	TS031	50	male	right	TS067	36	female	right
KN23	22	male	right	TS032	24	male	right				
KN25	22	female	right	TS036	27	female	right				
TS002	31	male	right	TS049	26	male	right				
TS003	30	female	right	TS053	29	male	right				
TS012	19	male	right	TS054	21	female	right				
TS014	26	male	right	TS059	35	female	right				
TS015	30	female	right	TS062	36	male	right				
TS016	26	female	right	TS068	25	male	right				
TS024	27	male	right	TS070	20	female	right				
TS025	27	male	right	TS073	44	female	right				
TS027	24	female	right								
TS028	26	male	right								
TS029	45	female	right								
TS030	27	male	right								
TS039	26	female	right								
TS041	25	female	left								
TS042	31	male	right								
TS047	28	female	right								
TS056	22	male	right								
TS060	24	female	right								
TS063	35	female	right								
TS071	27	female	right								
TS072	33	male	right								

Supplementary Table 2. (Tinnitus description)

			Tinnitus laterality		Tinnitus frequency & Intensity			
Tinnitus	Tinnitus Score	Right	Left	Both Sides Inner Head	Right		Left	
					Hz	dB	Hz	dB
T001	0	low	moderate	moderate	8000	15	8000	15
T002	3	low	low	---	10.000	6	10.000	4
T006	21	moderate	moderate	---	6000	27	6000	27
T009	7	---	moderate	---	---	---	8000	34
TN01	24	moderate	---	---	10.000	35	---	---
TN03	48	---	low	---	---	---	10.000	31
TN04	7	inaudible	inaudible	---	4000	5	4000	6
TN05	12	very low	inaudible	---	10.000	28	---	---
TN08	13	inaudible	inaudible	very low	10.000	13	10.000	10
TN10	7	inaudible	inaudible	inaudible	4000	12	8000	7
TN11	10	very low	very low	very low	6000	14	8000	7
TN16	19	high	low	moderate	8000	15	6000	3
TS004	22	---	low	---	---	---	10000	13
TS005	7	moderate	low	inaudible	3000	15	3000	21
TS008	19	---	moderate	---	---	---	500	10
TS010	13	??	??	low	8000	14	8000	18
TS017	18	inaudible	low	low	6000	18	6000	14
TS019	7	??	??	very low	2000	14	2000	11
TS021	10	low	low	inaudible	8000	13	8000	15
TS031	38	high	high	---	6000	34	6000	34
TS032	4	moderate	---	inaudible	8000	57	---	---
TS036	5	very low	---	inaudible	1500	15	---	---
TS049	15	low	very low	low	4000	5	4000	1
TS053	16	??	very low	??	6000	17	6000	17
TS054	9	low	very low	low	6000	11	8000	9
TS059	13	moderate	low	---	3000	23	3000	26
TS062	9	---	low	very low	---	---	8000	8
TS068	8	low	---	inaudible	4000	24	---	---
TS070	26	inaudible		inaudible	500	16	---	---
TS073	6	very low	---	---	125	11	---	---

Tinnitus+ Hyperacusis	Tinnitus Score	Right	Left	Both Sides Inner Head	Right		Left	
					Hz	dB	Hz	dB
TN12	18	low	very low	very low	1000	11	8000	22
TN13	9	---	very low	---	---	---	10000	5
TN17	31	very low	very low	very low	8000	8	8000	8
TN19	16	low	---	---	10000	20	---	---
TN20	30	low	high	inaudible	10000	21	1000	16
TN21	19	very low	very low	moderate	1000	11	750	13
TN22	45	moderate	moderate	---	6000	9	6000	10
TN23	31	low	low	---	1000	13	10000	9
TN25	23	low	moderate	inaudible	4000	15	4000	15
TN28	28	high	moderate	inaudible	8000	40	6000	23
TS020	35	---	moderate	---	---	---	6000	20
TS033	32	low	moderate	low	6000	10	6000	18
TS037	53	high	very low	low	4000	15	4000	20
TS040	27	low	moderate	---	8000	9	6000	11
TS044	23	---	inaudible	inaudible	---	---	750	5
TS048	32	??	??	moderate	8000	30	6000	9
TS050	22	low	low	---	6000	2	6000	1
TS057	18	moderate	moderate	inaudible	6000	9	6000	25
TS061	57	moderate	moderate	inaudible	10000	15	10000	16
TS067	23	very low	inaudible	inaudible	1500	10	4000	12

Supplementary Table 3. ABR latency and suprathreshold amplitude (*p*. Value)

ABR suprathreshold amplitude (75 dB nHL) (*p*. Value)

	T-group/Control		*T+H/Control*		*T-group/T+H*	
	Right	Left	Right	Left	Right	Left
Wave I	0.936	0.68	0.996	0.329	0.944	0.132
Wave III	0.186	0.379	0.221	<0.001***	0.006**	0.008**
Wave V	0.029*	0.050*	0.186	0.088	0.002**	<0.001***
Wave VI	0.004**	0.382	0.914	0.146	0.036*	0.040*

ABR suprathreshold amplitude (65 dB nHL) (*P*. Value)

	Tinnitus/Control		*T+H/Control*		*Tinnitus/T+H*	
	Right	Left	Right	Left	Right	Left
Wave I	0.546	0.978	0.447	0.268	0.291	0.28
Wave III	0.116	0.972	0.849	0.231	0.045*	0.254
Wave V	0.008**	0.462	0.774	0.355	0.095	0.030*
Wave VI	0.145	0.176	0.548	0.77	0.145	0.176

ABR Latency (*p*. Value)

		Tinnitus/Control		*T+H/Control*		*Tinnitus/T+H*	
		Right	Left	Right	Left	Right	Left
Wave I	75 dB	0.7	0.222	0.173	0.854	0.101	0.15
	65 dB	0.056	0.386	0.167	0.408	0.019*	0.050**
Wave III	75 dB	0.106	0.184	0.833	0.713	0.013*	0.018**
	65 dB	0.809	0.654	0.039**	0.012*	0.008**	0.004**
Wave V	75 dB	0.001**	<0.001***	0.312	0.395	0.050*	<0.001***
	65 dB	0.045*	0.003**	0.559	0.823	0.017*	0.002**
	55 dB	0.013*	0.063	0.271	0.157	0.007*	0.006*
	45 dB	<0.001***	0.027*	0.038**	0.058	<0.001***	0.011*
	35 dB	<0.001***	0.163	0.342	0.024**	<0.001***	0.011*
	25 dB	0.014*	0.079	0.018*	0.063	<0.001***	0.020*
Wave VI	75 dB	0.687	0.075	0.219	0.699	0.185	0.075
	65 dB	0.388	0.013*	0.115	0.039*	0.388	0.013*

Mean and SD of the ABR Wave V: gender comparison (75 dB nHL)

		Right ear			Left ear		
	group	Male	Female	*p*	Male	Female	*p*
Amplitude	Control	0.431 ± 0.16	0.486 ± 0.202	0.917	0.352 ± 0.201	0.435 ± 0.202	0.667
	T	0.385 ± 0.13	0.339 ± 0.125	0.987	0.329 ± 0.131	0.27 ± 0.112	0.965
	T+H	0.564 ± 0.196	0.517 ± 0.201	0.995	0.54 ± 0.25	0.47 ± 0.137	0.969
Latency	Control	5.306 ± 0.257	5.238 ± 0.209	0.951	5.24 ± 0.296	5.263± 0.212	>0.999
	T	5.501 ± 0.279	5.44 ± 0.292	0.991	5.506 ± 0.236	5.553 ± 0.19	0.996
	T+H	5.305 ± 0.138	5.35 ± 0.25	0.999	5.305 ± 0.218	5.313 ± 0.223	>0.999

Supplementary Table 4 Predefined ROIs for task evoked and resting state fMRI

Task evoked predefined ROIs

Brain Region	*MNI Coordinates (in mm)*			*Radius (in mm)*
(Brodmann Area)	*X*	*Y*	*Z*	
Subcortical Regions:				
CN-R/CN-L[1]	±10	-39	-45	3
SOC-R/SOC-L[1]	±13	-35	-41	3
IC-R/IC-L[1]	±6	-33	-11	3
MGB-R/MGB-L[1]	±17	-24	-2	3
Primary Auditory Cortex Regions:				
BA41-R/BA41-L[2]	49/-48	-13/-20	5/7	3
BA41A-R/BA41A-L[3]	53/-52	-3/-8	-2/2	3
BA41P-R/BA41P-L[2]	±40	-25/-30	10/11	3
BA42-R/BA42-L[4]	±64	-22	9	3
BA42A-R/BA42A-L[4]	±60	-18	10	3
BA42P-R/BA42P-L[4]	±56	-25	12	3
Sound Identification Regions:				
BA22A-R/BA22A-L[4]	±54	-6	-6	3
BA22P-R/BA22P-L[4]	±67	-27	3	3
BA21A-R/BA21A-L[4]	±66	-13	-5	3
BA21P-R/BA21P-L[4]	±66	-22	-5	3
Hipp-R/Hipp-L[5]	28/-29	-22/-19	-14/-15	3
BA13P-R/BA13P-L[2]	37/-38	-17/-19	6/5	3
Somatosensory/Pain Regions:				
PO_1-R/ PO_1-L[6]	±59	-23	25	3
PO_2-R/ PO_2-L[6]	±58	-14	18	3
DpIns-R/DpIns-L[6]	40/-41	-21	19	3
Mam.-Body-R/Mam.-Body-L[7]	4/-2	-12	-14	3

Resting state additional predefined ROIs

Brain Region	*MNI Coordinates (in mm)*			*Radius (in mm)*
(Brodmann Area)	*X*	*Y*	*Z*	
Emotional Regions				3
BA13A-R/BA13A-L[2]	32/-33	26/23	1/2	3
Amyg-R/Amyg-L[5]	21/-24	-1/0	-22/-21	3
Temporo-frontal Attentional Regions				
BA45-R/BA45-L[5]	46/-47	26/27	7/6	3
BA46-R/BA46-L[5]	43/-46	38	12/8	3
BA47-R/BA47-L[5]	38/-40	30/31	-12/-13	3
BA9M-R/BA9M-L[8]	±7	50	30	3
BA9DL-R/BA9DL-L[8]	±50	14	32	3

[11] Mühlau et al., 2006; [12] Amunts et al., 2020; [13] Amunts et al., 2019; [8] Hofmeier et al., 2018; [14] Lacadie et al., 2008; [15] Horing et al., 2019; [16] Lancaster et al., 2000; [17] Mai et al., 2016.

1. Hultcrantz M, Simonoska R, Stenberg AE. Estrogen and hearing: a summary of recent investigations. *Acta Otolaryngol*. Jan 2006;126(1):10-4. doi:10.1080/00016480510038617
2. Fischer A. Hyperakusis: Neues Screening-Instrument vorgestellt. journal article. *HNO Nachrichten*. June 01 2013;43(3):38-38. doi:10.1007/s00060-013-0111-x
3. Khalfa S, Dubal S, Veuillet E, Perez-Diaz F, Jouvent R, Collet L. Psychometric normalization of a hyperacusis questionnaire. *ORL J Otorhinolaryngol Relat Spec*. Nov-Dec 2002;64(6):436-42. doi:10.1159/000067570
4. Nelting M, Rienhoff NK, Hesse G, Lamparter U. [The assessment of subjective distress related to hyperacusis with a self-rating questionnaire on hypersensitivity to sound]. *Laryngorhinootologie*. May 2002;81(5):327-34. Die Erfassung des subjektiven Leidens unter Hyperakusis mit einem Selbstbeurteilungsbogen zur Gerauschuberempfindlichkeit (GUF). doi:10.1055/s-2002-28342
5. Hiller W, Goebel G, Rief W. Reliability of self-rated tinnitus distress and association with psychological symptom patterns. *Br J Clin Psychol*. May 1994;33 (Pt 2):231-9.
6. Goebel G, Hiller W. [The tinnitus questionnaire. A standard instrument for grading the degree of tinnitus. Results of a multicenter study with the tinnitus questionnaire]. *HNO*. Mar 1994;42(3):166-72. Tinnitus-Fragebogen (TF). Standardinstrument zur Graduierung des Tinnitusschweregrades. Ergebnisse einer Multicenterstudie mit dem Tinnitus-Fragebogen (TF).
7. Biesinger E, Heiden C, Greimel V, Lendle T, Hoing R, Albegger K. [Strategies in ambulatory treatment of tinnitus]. *HNO*. Feb 1998;46(2):157-69. Strategien in der ambulanten Behandlung des Tinnitus. doi:10.1007/s001060050215
8. Hofmeier B, Wolpert S, Aldamer ES, et al. Reduced sound-evoked and resting-state BOLD fMRI connectivity in tinnitus. *Neuroimage Clin*. 2018;20:637-649. doi:10.1016/j.nicl.2018.08.029
9. Guimaraes AR, Melcher JR, Talavage TM, et al. Imaging subcortical auditory activity in humans. *Hum Brain Mapp*. 1998;6(1):33-41.
10. Wobbrock JO, Findlater L, Gergle D, Higgins JJ. The Aligned Rank Transform for nonparametric factorial analyses using only ANOVA procedures. presented at: CHI 2011 - 29th Annual CHI Conference on Human Factors in Computing Systems-Proceedings; May 07 2011 - May 12 2011 2011; Vancouver, BC, Canada.
11. Muhlau M, Rauschecker JP, Oestreicher E, et al. Structural brain changes in tinnitus. *Cereb Cortex*. Sep 2006;16(9):1283-8. doi:10.1093/cercor/bhj070
12. Amunts K, Mohlberg H, Bludau S, Caspers S, Eickhoff SB, Pieperhoff P. Data from: Whole-brain parcellation of the Julich-Brain Cytoarchitectonic Atlas 2020;2.0. doi:10.25493/TAKY-64D
13. Amunts K, Eickhoff SB, Caspers S, Bludau S, Mohlberg H. Data from: Whole-brain parcellation of the Julich-Brain Cytoarchitectonic Atlas 2019;1.18. doi:10.25493/8EGG-ZAR
14. Lacadie CM, Fulbright RK, Rajeevan N, Constable RT, Papademetris X. More accurate Talairach coordinates for neuroimaging using non-linear registration. *Neuroimage*. Aug 15 2008;42(2):717-25. doi:10.1016/j.neuroimage.2008.04.240
15. Horing B, Sprenger C, Buchel C. The parietal operculum preferentially encodes heat pain and not salience. *PLoS Biol*. Aug 2019;17(8):e3000205. doi:10.1371/journal.pbio.3000205
16. Lancaster JL, Woldorff MG, Parsons LM, et al. Automated Talairach atlas labels for functional brain mapping. *Hum Brain Mapp*. Jul 2000;10(3):120-31. doi:10.1002/1097-0193(200007)10:3<120::aid-hbm30>3.0.co;2-8

17. Mai JK, Majtanik M, Paxinos G. *Atlas of the Human Brain*. vol 4. Academic Press is an imprint of Elsevier; 2016.
